# Author Correction: Global hotspots of salt marsh change and carbon emissions

**DOI:** 10.1038/s41586-023-06666-5

**Published:** 2023-10-09

**Authors:** Anthony D. Campbell, Lola Fatoyinbo, Liza Goldberg, David Lagomasino

**Affiliations:** 1 Biospheric Sciences Laboratory, National Aeronautics and Space Administration (NASA) Goddard Space Flight Center, Greenbelt, MD USA; 2 NASA Postdoctoral Program, Oak Ridge Associated Universities, Oak Ridge, TN USA; 3 GESTAR II, University of Maryland, Baltimore County, Baltimore, MD USA; 4 Earth System Science Interdisciplinary Center, University of Maryland, College Park, MD USA; 5 Integrated Coastal Programs, East Carolina University, Wanchese, NC USA

**Keywords:** Natural hazards, Environmental impact, Climate-change impacts

Correction to: *Nature* 10.1038/s41586-022-05355-z Published online 30 November 2022

In the version of this article initially published, Supplementary Table 12 had incorrect duplicate values in the SOCS 2019 columns. This table has now been updated.

