# Supplementary material for: Global hotspots of salt marsh change and carbon emissions
Source: Nature. 2022 Nov 30;612(7941):701–6. doi: 10.1038/s41586-022-05355-z (PMC9771810; doi:10.1038/s41586-022-05355-z)
Supplement: Supplementary file 1 — Thirteen supplementary tables, including salt marsh loss and gain by epoch and country, panel analysis of change drivers, accuracy assessments, threshold sensitivity and SOCS estimates for 2019. Brief supplemental notes on the accuracy assessment, mangrove/marsh overlap in Oceania and salt marsh extent in China. [file 41586_2022_5355_MOESM1_ESM.pdf]

nature portfolio

https://doi.org/10.1038/s41586-022-05355-z

Supplementary information

Global hotspots of salt marsh change and carbon emissions

In the format provided by the
authors and unedited

Supplemental Information

Campbell et al. Global hotspots of salt marsh change and carbon emissions

Table S1. Salt marsh loss and gain anomalies for each period by country in hectares. Excluding mangrove areas in Australia and New Zealand and updated extent estimates for China, Taiwan, and Vietnam.

Table S2. A panel analysis of the Contiguous United States (CONUS) comparing salt marsh loss and gains to local sea level change trends, hurricane landfalls and category, urbanization, and change within 100 m of the salt marsh.

Table S3. Salt marsh change accuracy assessment for 2000-2004 for a total of 3150 points distributed globally. Reference category refers to the classes (gain, loss, or stable) as determined with the high-resolution accuracy assessment. Salt marsh change anomaly category classes (gain, loss, or stable) are determined by the Normalized Difference Vegetation Index (NDVI) anomaly analysis.

Table S4. Salt marsh change accuracy assessment for 2005-2009 for a total of 3150 points distributed globally. Reference category refers to the classes (gain, loss, or stable) as determined with the high-resolution accuracy assessment. Salt marsh change anomaly category classes (gain, loss, or stable) are determined by the NDVI anomaly analysis.

Table S5. Salt marsh change accuracy assessment for 2010-2014 for a total of 3150 points distributed globally. Reference category refers to the classes (gain, loss, or stable) as determined with the high-resolution accuracy assessment. Salt marsh change anomaly category classes (gain, loss, or stable) are determined by the NDVI anomaly analysis.

Table S6. Salt marsh change accuracy assessment for 2015-2019 for a total of 3150 points distributed globally. Reference category refers to the classes (gain, loss, or stable) as determined with the high-resolution accuracy assessment. Salt marsh change anomaly category classes (gain, loss, or stable) are determined by the NDVI anomaly analysis.

Table S7. Salt marsh accuracy assessment conducted with 6845 points distributed across the globe. Reference (salt marsh or other land cover) being the results of high-resolution accuracy assessment. Salt marsh extent (Mcowen) were the land cover class as identified by the Mcowen salt marsh layer[1].

Table S8. Salt marsh recovery accuracy assessment for all recovery periods for a total of 2000 points across two USA watersheds. Reference refers to the class determined with the high-resolution accuracy assessment. Salt marsh recovery was determined by the NDVI anomaly analysis.

Table S9. Salt marsh change threshold assessment of 1278 points split between loss and gain across 6 threshold levels of 0.15-0.20 NDVI.

Table S10. Beta regression results comparing percent loss for each period to the mapping year.

Table S11. Beta regression results comparing percent gain for each period to the mapping year.

Table S12. Country level net SOCS change from 2000-2019 and total 2019 soil organic carbon stock (SOCS) and confidence interval.

Table S13. References accessed through the Coastal Carbon Atlas and used to calculate emergent vegetation soil organic carbon stock.

References

Table S1. Salt marsh loss and gain anomalies for each period by country in hectares. Excluding mangrove areas in Australia and New Zealand and updated extent estimates for China, Taiwan, and Vietnam.

Country	Loss 2000-2004	Loss 2005-2009	Loss 2010-2014	Loss 2015-2019	Gain 2000-2004	Gain 2005-2009	Gain 2010-2014	Gain 2015-2019
Albania	81.68	81.29	241.45	270.73	60.07	109.89	116.00	100.10
Argentina	7738.41	637.75	2113.60	4150.13	524.00	293.69	232.44	300.60
Australia	3125.63	3067.42	4734.68	3525.55	0.78	2518.79	7836.41	6682.32
Belgium	0.00	0.00	5.88	1.31	0.00	0.00	0.00	0.00
Brazil	9.51	14.16	29.53	39.01	8.70	4.39	8.17	14.61
Canada	395.47	240.75	567.38	606.50	421.06	517.31	610.44	719.72
Chile	14.28	3.25	1.77	5.79	33.57	26.30	39.77	36.51
China	5748.27	7601.90	7851.75	6532.69	6691.05	11592.44	4879.47	4551.71
Cyprus	486.51	8.44	429.25	30.40	0.00	0.00	2.00	3.22
Estonia	1.93	0.78	1.75	4.65	0.51	1.98	0.32	0.18

Finland	1164.70	657.84	106.30	200.59	40.76	76.07	226.42	212.06
France	1792.65	887.52	1362.69	991.49	241.03	600.04	591.41	951.40
Greece	9.77	3.37	14.17	15.41	1.03	13.27	34.60	40.17
Iceland	5.54	1.88	6.55	37.77	1.67	137.91	70.18	48.21
Ireland	357.23	123.42	60.62	51.47	30.83	26.86	55.60	36.55
Italy	620.92	1177.91	2455.58	4751.14	183.17	631.54	1033.02	688.00
Latvia	1.34	0.46	4.71	2.38	0.25	1.64	3.10	1.64
Madagascar	158.56	220.47	76.73	119.95	54.91	14.42	35.46	68.87
Mexico	3375.15	2174.88	2721.83	3116.92	443.56	759.88	824.50	1888.49
Netherlands	320.33	105.17	179.20	276.21	74.60	429.86	212.16	217.51
New Zealand	580.84	227.98	566.50	241.53	0.00	95.16	134.64	166.85
Peru	340.05	147.20	235.50	147.28	745.88	646.78	521.26	440.31
Portugal	267.22	1123.42	1090.32	713.85	15.83	52.69	115.21	53.44

Russia	117.76	11206.30	13384.24	27940.51	1.06	1136.06	1204.13	555.68
South Africa	122.79	23.00	52.75	62.36	139.53	528.31	235.72	48.33
Spain	2245.75	759.70	1511.67	1777.92	832.26	2847.87	1640.25	2837.39
Sweden	280.81	88.69	46.94	91.09	4.14	6.24	17.12	9.60
Taiwan	0.00	1.45	2.57	2.73	0.00	0.48	0.64	0.64
Turkey	1084.02	391.95	763.90	1278.08	140.61	891.80	1226.23	882.44
United Arab Emirates	5.41	3.12	15.15	3.51	2.91	15.33	11.98	34.11
United Kingdom	716.84	266.72	406.70	426.04	189.82	1783.73	1003.09	1027.39
Uruguay	53.25	20.95	35.29	32.52	4.69	4.26	16.33	19.10
USA	27442.86	34471.76	22627.60	24602.31	7648.77	7577.86	9888.47	19658.16
Vietnam	29.38	42.08	23.76	14.19	0.33	1.65	2.64	5.78
Croatia	6.27	5.50	7.68	4.35	0.00	0.03	1.73	1.54

Denmark	1034.30	213.00	596.60	498.29	40.75	6.15	45.88	57.12
Germany	198.12	64.31	65.59	117.24	22.01	9.76	111.70	38.49
Montenegro	0.00	0.00	0.00	0.45	0.00	0.00	0.00	0.00
Slovenia	34.87	18.88	6.66	8.19	0.00	0.00	0.38	0.00

Table S2. A panel analysis of the Contiguous United States (CONUS) comparing salt marsh loss and gains to local sea level change trends, hurricane landfalls and category, urbanization, and change within 100 m of the salt marsh.

	**Salt marsh gain**			**Salt marsh loss**		
Predictors	*Estimates*	*CI*	*p*	*Estimates*	*CI*	*p*
Loss 100 m	0.02	0.00 – 0.05	**0.044**	0.77	0.70 – 0.84	**<0.001**
Gain 100 m	1.35	1.19 – 1.50	**<0.001**	-2.02	-2.49 – -1.56	**<0.001**
Urbanization	0.00	-0.00 – 0.00	0.306	0.00	-0.00 – 0.00	0.108
Hurricanes	-5.92	-23.95 – 12.11	0.521	58.32	2.86 – 113.78	**0.041**
LSLC trend	-23.07	-35.98 – -10.16	**0.001**	-3.61	-43.33 – 36.12	0.859
Observations	180			180		
R^2 / R^2 adjusted	0.740 / 0.642			0.802 / 0.727		

Accuracy Assessments

Three accuracy assessments were conducted to verify the results and calculate robust error estimates for the analysis. Confidence intervals were calculated adapting previous methods[1]. Confidence intervals were calculated as percent error and applied to our original estimates of change due to the limited regional data and difficulty ascertaining true change with limited high-resolution data in some regions (Table S3-S6). The salt marsh extent accuracy demonstrates that while the extent layer includes majority salt marsh, error of both omission and commission were common (Table S7). We assessed the accuracy of the extent with points from China, England, USA, Australia, Mexico, and Peru for 2019. In remote areas of the assessment relied on limited imagery, and most recent images were used to determine salt marsh presence. Recovery detection was accurate achieving an overall accuracy of 96.1 (n =2000; Table S8). Additionally, the 0.2 NDVI magnitude threshold was assessed across 1278 points. Each threshold was resampled to resemble the distribution of the data resulting in gain (n=378) and loss (n = 248). The 0.20 threshold performed best in the accuracy assessment (Table S9).

Table S3. Salt marsh change accuracy assessment for 2000-2004 for a total of 3150 points distributed globally. Reference category refers to the classes (gain, loss, or stable) as

determined with the high-resolution accuracy assessment. Salt marsh change anomaly category classes (gain, loss, or stable) are determined by the Normalized Difference Vegetation Index (NDVI) anomaly analysis.

		**Reference**			
		Gain	Loss	Stable	Producers Accuracy
**Salt marsh change anomaly**	Gain	972	28	6	96.6
	Loss	23	947	33	94.4
	Stable	55	75	1011	88.6
	Users Accuracy	92.6	90.2	96.3	Overall Accuracy: 93.0

Table S4. Salt marsh change accuracy assessment for 2005-2009 for a total of 3150 points distributed globally. Reference was the class (gain, loss, or stable) as determined with the high-resolution accuracy assessment. Salt marsh change anomaly was the class (gain, loss, or stable) as determined by the NDVI anomaly analysis.

		**Reference**			
		Gain	Loss	Stable	Producers Accuracy
**Salt marsh change**	Gain	931	27	8	96.4
	Loss	34	956	30	93.7
	Stable	85	67	1012	86.9
	Users Accuracy	88.7	91.0	96.4	Overall Accuracy: 92.0

Table S5. Salt marsh change accuracy assessment for 2010-2014 for a total of 3150 points distributed globally. Reference was the class (gain, loss, or stable) as determined with the high-resolution accuracy assessment. Salt marsh change anomaly was the class (gain, loss, or stable) as determined by the NDVI anomaly analysis.

		**Reference**			
		Gain	Loss	Stable	Producers Accuracy
**Salt marsh change**	Gain	893	24	14	95.9
	Loss	48	955	25	92.9
	Stable	109	71	1011	84.9
	Users Accuracy	85.0	91.0	96.3	Overall Accuracy: 90.8

Table S6. Salt marsh change accuracy assessment for 2015-2019 for a total of 3150 points distributed globally. Reference was the class (gain, loss, or stable) as determined with the high-resolution accuracy assessment. Salt marsh change anomaly was the class (gain, loss, or stable) as determined by the NDVI anomaly analysis.

		Reference			
**Salt marsh change**		Gain	Loss	Stable	Producers Accuracy
	Gain	937	27	10	96.2
	Loss	20	899	25	95.2
	Stable	93	124	1015	82.4
	Users Accuracy	89.2	85.6	96.7	Overall Accuracy: 90.5

Table S7. Salt marsh accuracy assessment conducted with 6845 points distributed across the globe. Reference (salt marsh or other land cover) being the results of high-resolution accuracy assessment. Salt marsh extent (Mcowen; salt marsh or other land cover) were the land cover class as identified by the Mcowen salt marsh layer[1].

		Reference		
**Salt marsh extent (Mcowen)**		Other Land Cover	Salt marsh	Producers Accuracy
	Other Land Cover	3213	518	86.1
	Salt marsh	209	2905	93.3
	Users Accuracy	93.9	84.9	Overall Accuracy: 89.4

Table S8. Salt marsh recovery accuracy assessment for all recovery periods for a total of 2000 points across two USA watersheds. Reference refers to the class determined with the high-resolution accuracy assessment. Salt marsh recovery was determined by the NDVI anomaly analysis.

		Reference		
**Salt marsh Recovery**		Loss	Recovery	Producers Accuracy
	Loss	959	41	96.2
	Recovery	38	962	95.9
	Users Accuracy	95.9	96.2	Overall Accuracy: 96.1

Table S9. Salt marsh change threshold assessment of 1278 points split between loss and gain across 11 threshold magnitudes of 0.15-0.25 NDVI.

Threshold Magnitude	Gain Accuracy	Loss Accuracy
0.15	0.21	0.59
0.16	0.48	0.70
0.17	0.65	0.79
0.18	0.80	0.88
0.19	0.89	0.93
0.20	0.95	0.96
0.21	0.88	0.92
0.22	0.83	0.81
0.23	0.81	0.73
0.24	0.79	0.67
0.25	0.78	0.52

China reclassification with ancillary data

Ancillary data were utilized to investigate reclamation within Asia. Reclamation was investigated with 30 m LCLU data created in 2015[3]. Reclamation will often result in a gain anomaly, i.e., increase in NDVI due to the conversion from salt marsh to upland land use. LCLU data were utilized to determine overlap between gain anomalies and likely reclamation drivers, i.e., agriculture and urban LCLU. These areas were reclassified as salt marsh loss. This approach was relatively conservative, as all upland classes could have been included as loss anomalies.

Salt marsh change was analyzed in seven global regions, of which Asia had the highest combined losses and gains. In China, a single watershed Yangtze River Delta Estuary (4060009290), was responsible for approximately 38% of all loss. When visually inspected, it was clear these losses corresponded with a decrease in total suspended solids in the Yangtze River. The misclassification of water as change was evident in only this watershed from 2010-2019, but inclusion of water was evident within the salt marsh extent accuracy assessment which had an overall accuracy of 55.3%. Misclassification of water was a major driver of this uncertainty in the Chinese wetland extent, e.g., in 2015, 84% of the area within the salt marsh extent was classified as water. While some misclassification of salt marsh as water is expected, this magnitude was likely due to differing definitions in the regional classification. China had two clear salt marsh extents one which included extensive areas of water. The water polygons were removed from the extent and a new salt marsh extent accuracy assessment and

change analysis were conducted. Utilizing the new extent only 31% of area within the salt marsh extent was water and the classification accuracy went up to 83.7% overall accuracy. Reclamation was prevalent in China, e.g., agricultural areas, amounted to 39% of all salt marsh gains in China compared to the USA's 0.53 % of gains. Prior to this reclassification 44% of salt marsh within China, Taiwan, and Vietnam experienced gain anomalies. These updated change estimates are in Table S1 and used throughout the paper.

In general, the starting salt marsh extent underestimated loss in China, e.g., the Yellow River delta lost approximately 15,000 ha from 2000-2004[4], and only 155 ha was picked up by this analysis due to our studies limited salt marsh extent. In 2000, China was estimated to have a maximum of 2,100,000 ha of coastal wetlands in 2000 and a lose rate of 1.024% yr^{-1} since 1950[5]. If our observed loss rate of 1.34% for Asia were applied to this extent global salt marsh emissions would double with an additional 0.0144 Pg CO_2e yr^{-1} from China alone. An updated map of salt marsh in China is needed to reduce uncertainty of this analysis. Recent analysis of salt marsh change in China from 1985 to 2019 has demonstrated limited change in total marsh extent from 2000-2019[6]. Both that study and ours demonstrate very low net change rates due to prograding coasts of the region.

Baseline mapping year uncertainty

To assess the effect of mapping year on our change analysis we used the National Wetland Inventory, which comprised our salt marsh extent for the United States. The National Wetland Inventory's ancillary data showing image acquisition was converted into a raster. The raster was clipped to the salt marsh extent. This image year layer was then used to calculate an average baseline mapping year for each watershed.

A later mapping year was more likely to have less loss for all years except 2015-2019, before which almost all mapping had occurred (Table S10). In comparison, percent gain was not as impacted by the mapping year, with only the 2015-2019 period having any significant relationship to the mapping year (Table S11). Losses were more impacted by mapping year than gains suggesting that our method slightly underestimated global loss.

Table S10. Beta regression results comparing percent loss for each period to the mapping year.

	Salt marsh loss (2000-2004)	Salt marsh loss (2005-2009)	Salt marsh loss (2010-2014)	Salt marsh loss (2015-2019)
(Intercept)	92.68 ***	39.10 **	40.27 **	-17.20
	(15.79)	(14.55)	(12.97)	(13.59)
Mapping Year	-0.05 ***	-0.02 **	-0.02 ***	0.01
	(0.01)	(0.01)	(0.01)	(0.01)
(phi)	25.73 ***	73.82 ***	75.25 ***	86.46 ***
	(4.81)	(12.87)	(12.06)	(13.64)
# obs	96	95	100	98
Pseudo R^2	0.33	0.13	0.12	0.01

Residual	93.00	92.00	97.00	95.00

*** p < 0.001; ** p < 0.01; * p < 0.05.

Table S11. Beta regression results comparing percent gain for each period to the mapping year.

	Salt marsh gain (2000-2004)	Salt marsh gain (2005-2009)	Salt marsh gain (2010-2014)	Salt marsh gain (2015-2019)
(Intercept)	14.91	22.73	9.76	28.71
	(17.17)	(15.92)	(15.77)	(15.30)
Mapping Year	-0.01	-0.01	-0.01	-0.02 *
	(0.01)	(0.01)	(0.01)	(0.01)
(phi)	14.72 ***	41.51 ***	57.31 ***	37.11 ***
	(3.21)	(7.86)	(10.64)	(6.60)
# obs	90	94	95	97
Pseudo R^2	0.03	0.04	0.01	0.05
Residual	87.00	91.00	92.00	94.00

*** p < 0.001; ** p < 0.01; * p < 0.05.

Mapping year impacted loss anomalies more than gain anomalies, likely due to loss being more prevalent and easier to exclude when mapping. The mapping date's impact clarifies the need for a single date global salt marsh map, which would reduce the uncertainty in monitoring. Our study's neighboring change metrics captured these earlier salt marsh losses but could not be isolated from other non-salt marsh related changes. Including areas neighboring the salt marsh may minimize the effect of mapping date but would introduce new sources of error into the analysis. Change neighboring the salt marsh within the CONUS was low in 2000-2004 compared to subsequent years e.g., 60% less loss anomalies than the second lowest loss period 2010-2014. Gradual change processes were common i.e., erosion, pond expansion and channel enlargement. These changes take time to alter the NDVI of a single pixel. Both gradual change processes and the impact of mapping date resulted in a low amount of observed loss in 2000-2004.

Oceania mangrove encroachment analysis

In Australia, mangrove encroachment was investigated to determine where mangrove extent coincided spatially with salt marsh loss or gain. The analysis used the Global Mangrove Watch 2016 layer[7]. Salt marsh change anomalies in Australia were analyzed in relation to mangrove encroachment to determine its role in the observed salt marsh change. We hypothesized that a large proportion of gain anomalies were due to mangrove encroachment. In total, 36% of gain anomalies were within the mangrove extent. In Oceania 139 of the 190 watersheds analyzed had mangroves within them, and the ten watersheds with the greatest mangrove encroachment comprised 36% of all mangrove derived gains. Excluding mangrove encroachment reduced gains from 27,099 to 17,263 ha. In comparison, the mangrove extent only accounted for 880 hectares of loss anomalies. If the mangrove gain area is removed from

the Australian change metrics, then Australian salt marsh had slightly more gains than losses – however, if gains overlapping with mangroves are considered a loss, then Australian mangrove regions experienced a net loss i.e., gains of 17263 and losses of 25109 ha. However, it is important to note that these shifts are likely to be beneficial to carbon sequestration, and therefore were not included in our carbon analysis.

Table S12. Country level net SOCS change from 2000-2019 and total 2019 soil organic carbon stock (SOCS) and confidence interval in Teragrams.

	Loss in top 30 cm of SOCS 2000-2019 (Tg)			*SOCS 2019* (Tg)		
Country	*Mean*	*Quantile 0.05*	*Quantile 0.95*	*Mean*	*Quantile 0.05*	*Quantile 0.95*
United States of America	9.54977	1.55758	30.52779	135.579	22.8466	383.541
Australia	0.70945	0.10936	1.96146	63.2319	9.25978	177.971
Russia	3.71114	0.62884	10.80895	48.1413	8.28685	135.342
Canada	0.14703	0.03471	0.33528	10.0063	2.36671	22.8454
Mexico	0.36998	0.06432	1.05132	8.02929	1.60629	20.8026
Argentina	0.66451	0.08909	1.96318	5.56434	0.83855	14.8139
Spain	0.30142	0.07790	0.72837	5.41973	1.3437	13.0548
France	0.26545	0.06883	0.70342	4.49902	1.33564	10.7867
United Kingdom	0.13555	0.03662	0.32508	4.28535	1.29427	9.52856
China	1.25945	0.26061	3.10698	2.14769	0.4279	5.37251
Italy	0.51848	0.12865	1.34481	2.07231	0.53343	5.42748
New Zealand	0.14433	0.02417	0.36091	1.84727	0.323	4.49582
Denmark	0.15730	0.03545	0.36146	1.84555	0.23248	4.57176
Turkey	0.21829	0.03581	0.56797	1.67452	0.36138	4.59434
Germany	0.03069	0.00755	0.07417	1.15789	0.29375	2.37232

Finland	0.20476	0.04859	0.48093	1.11818	0.24893	2.4871
Portugal	0.22830	0.04449	0.62621	0.98639	0.21042	2.6133
Peru	0.03213	0.00519	0.09389	0.96022	0.14736	2.88806
Netherlands	0.05026	0.01378	0.12331	0.93954	0.25078	2.29788
Ireland	0.05300	0.02118	0.11452	0.84738	0.3213	1.77689
Sweden	0.04552	0.01045	0.10973	0.4528	0.09231	1.0697
Brazil	0.00638	0.00135	0.01848	0.42426	0.08567	1.15291
South Africa	0.01228	0.00163	0.04062	0.38832	0.05764	1.19102
Albania	0.04997	0.00885	0.15427	0.25411	0.04255	0.83271
Iceland	0.00464	0.00081	0.01099	0.25009	0.04091	0.60116
Madagascar	0.02073	0.00287	0.06209	0.21004	0.02989	0.60238
Uruguay	0.01075	0.00200	0.02443	0.17416	0.03301	0.40415
United Arab Emirates	0.00083	0.00010	0.00210	0.12924	0.01523	0.32025
Cyprus	0.05535	0.00829	0.16499	0.05531	0.00829	0.16297
Croatia	0.00159	0.00043	0.00301	0.04051	0.00983	0.07339
Greece	0.00278	0.00065	0.00714	0.04011	0.00874	0.10129
Chile	0.00118	0.00016	0.00349	0.03279	0.00507	0.10202
Estonia	0.00070	0.00015	0.00182	0.02711	0.00563	0.06898
Vietnam	0.00607	0.00135	0.01476	0.01272	0.00276	0.03082

Montenegro	0.00003	0.00001	0.00008	0.0066	0.0016	0.01196
Latvia	0.00061	0.00011	0.00154	0.00607	0.0015	0.01542
Slovenia	0.00453	0.00125	0.00779	0.00514	0.00125	0.00931
Belgium	0.00040	0.00011	0.00089	0.00316	0.00094	0.00701
Taiwan	0.00028	0.00005	0.00057	0.00082	0.00016	0.00164

Table S13: References accessed through the Coastal Carbon Atlas and used to calculate emergent vegetation soil organic carbon stock.

**Row**	Reference	Core depth represents deposit depth
1	Ensign, S.H., Noe, G.B., Hupp, C.R. and Skalak, K.J., 2015. Head-of-tide bottleneck of particulate material transport from watersheds to estuaries. *Geophysical Research Letters*, *42*(24), pp.10-671.	
2	Jones, M.C., Bernhardt, C.E., Krauss, K.W. and Noe, G.B., 2017. The impact of late Holocene land use change, climate variability, and sea level rise on carbon storage in tidal freshwater wetlands on the southeastern United States coastal plain. *Journal of Geophysical Research: Biogeosciences*, *122*(12), pp.3126-3141.	
3	Abbott, K.M., Elsey-Quirk, T. and DeLaune, R.D., 2019. Factors influencing blue carbon accumulation across a 32-year chronosequence of created coastal marshes. *Ecosphere*, *10*(8), p.e02828.	
4	Arriola, J.M. and Cable, J.E., 2017. Variations in carbon burial and sediment accretion along a tidal creek in a Florida salt marsh. *Limnology and Oceanography*, *62*(S1), pp.S15-S28.	
5	Cochran, J.K., Hirschberg, D.J., Wang, J. and Dere, C., 1998. Atmospheric deposition of metals to coastal waters (Long Island Sound, New York USA): evidence from saltmarsh deposits. *Estuarine, Coastal and Shelf Science*, *46*(4), pp.503-522.	
6	Neubauer, S.C., Anderson, I.C., Constantine, J.A. and Kuehl, S.A., 2002. Sediment deposition and accretion in a mid-Atlantic (USA) tidal freshwater marsh. *Estuarine, Coastal and Shelf Science*, *54*(4), pp.713-727.	
7	Thom, R.M., 1992. Accretion rates of low intertidal salt marshes in the Pacific Northwest. *Wetlands*, *12*(3), pp.147-156.	
8	Drake, K., Halifax, H., Adamowicz, S.C. and Craft, C., 2015. Carbon sequestration in tidal salt marshes of the Northeast United States. *Environmental Management*, *56*(4), pp.998-1008.	
9	Pastore, M.A., Megonigal, J.P. and Langley, J.A., 2017. Elevated CO2 and nitrogen addition accelerate net carbon gain in a brackish marsh. *Biogeochemistry*, *133*(1), pp.73-87.	

10	Callaway, J.C., Borgnis, E.L., Turner, R.E. and Milan, C.S., 2012. Carbon sequestration and sediment accretion in San Francisco Bay tidal wetlands. *Estuaries and Coasts*, *35*(5), pp.1163-1181.	
11	Watson, E.B. and Byrne, R., 2013. Late Holocene Marsh Expansion in Southern San Francisco Bay, California. *Estuaries and Coasts*, *36*(3), pp.643-653.	*
12	Lagomasino, D., Corbett, D.R. and Walsh, J.P., 2013. Influence of wind-driven inundation and coastal geomorphology on sedimentation in two microtidal marshes, Pamlico River Estuary, NC. *Estuaries and Coasts*, *36*(6), pp.1165-1180.	
13	Doughty, C.L., Langley, J.A., Walker, W.S., Feller, I.C., Schaub, R. and Chapman, S.K., 2016. Mangrove range expansion rapidly increases coastal wetland carbon storage. *Estuaries and Coasts*, *39*(2), pp.385-396.	
14	Noe, G.B., Hupp, C.R., Bernhardt, C.E. and Krauss, K.W., 2016. Contemporary deposition and long-term accumulation of sediment and nutrients by tidal freshwater forested wetlands impacted by sea level rise. *Estuaries and Coasts*, *39*(4), pp.1006-1019.	
15	Radabaugh, K.R., Moyer, R.P., Chappel, A.R., Powell, C.E., Bociu, I., Clark, B.C. and Smoak, J.M., 2018. Coastal blue carbon assessment of mangroves, salt marshes, and salt barrens in Tampa Bay, Florida, USA. *Estuaries and Coasts*, *41*(5), pp.1496-1510.	*
16	Elsey-Quirk, T., Seliskar, D.M., Sommerfield, C.K. and Gallagher, J.L., 2011. Salt marsh carbon pool distribution in a mid-Atlantic lagoon, USA: sea level rise implications. *Wetlands*, *31*(1), pp.87-99.	
17	Boyd, B.M. and Sommerfield, C.K., 2016. Marsh accretion and sediment accumulation in a managed tidal wetland complex of Delaware Bay. *Ecological Engineering*, *92*, pp.37-46.	
18	Nolte, S., Müller, F., Schuerch, M., Wanner, A., Esselink, P., Bakker, J.P. and Jensen, K., 2013. Does livestock grazing affect sediment deposition and accretion rates in salt marshes?. *Estuarine, Coastal and Shelf Science*, *135*, pp.296-305.	
19	Kulawardhana, R.W., Feagin, R.A., Popescu, S.C., Boutton, T.W., Yeager, K.M. and Bianchi, T.S., 2015. The role of elevation, relative sea-level history and vegetation transition in determining carbon distribution in Spartina alterniflora dominated salt marshes. *Estuarine, Coastal and Shelf Science*, *154*, pp.48-57.	
20	Hill, T.D. and Anisfeld, S.C., 2015. Coastal wetland response to sea level rise in Connecticut and New York. *Estuarine, Coastal and Shelf Science*, *163*, pp.185-193.	
21	Boyd, B.M., Sommerfield, C.K. and Elsey-Quirk, T., 2017. Hydrogeomorphic influences on salt marsh sediment accumulation and accretion in two estuaries of the US Mid-Atlantic coast. *Marine Geology*, *383*, pp.132-145.	
22	Gerlach, M.J., Engelhart, S.E., Kemp, A.C., Moyer, R.P., Smoak, J.M., Bernhardt, C.E. and Cahill, N., 2017. Reconstructing Common Era relative sea-level change on the Gulf Coast of Florida. *Marine Geology*, *390*, pp.254-269.	*
23	Johnson, B.J., Moore, K.A., Lehmann, C., Bohlen, C. and Brown, T.A., 2007. Middle to Late Holocene fluctuations of C3 and C4 vegetation in a northern New England salt marsh, Sprague Marsh, Phippsburg Maine. *Organic Geochemistry*, *38*(3), pp.394-403.	
24	Kemp, A.C., Sommerfield, C.K., Vane, C.H., Horton, B.P., Chenery, S., Anisfeld, S. and Nikitina, D., 2012. Use of lead isotopes for developing chronologies in recent salt-marsh sediments. *Quaternary Geochronology*, *12*, pp.40-49.	

25	Krauss, K.W., Noe, G.B., Duberstein, J.A., Conner, W.H., Stagg, C.L., Cormier, N., Jones, M.C., Bernhardt, C.E., Graeme Lockaby, B., From, A.S. and Doyle, T.W., 2018. The role of the upper tidal estuary in wetland blue carbon storage and flux. *Global Biogeochemical Cycles*, *32*(5), pp.817-839.	
26	Kauffman, J.B., Giovanonni, L., Kelly, J., Dunstan, N., Borde, A., Diefenderfer, H., Cornu, C., Janousek, C., Apple, J. and Brophy, L., 2020. Total ecosystem carbon stocks at the marine-terrestrial interface: Blue carbon of the Pacific Northwest Coast, United States. *Global Change Biology*, *26*(10), pp.5679-5692.	
27	Keshta, A.E.S.S., 2017. *Hydrology, Soil Redox, and Pore-Water Iron Regulate Carbon Cycling in Natural and Restored Tidal Freshwater Wetlands in the Chesapeake Bay, Maryland, USA* (Doctoral dissertation, University of Maryland, College Park).	
28	Orson, R.A., Simpson, R.L. and Good, R.E., 1990. Rates of sediment accumulation in a tidal freshwater marsh. *Journal of Sedimentary Research*, *60*(6), pp.859-869.	
29	Weis, D.A., Callaway, J.C. and Gersberg, R.M., 2001. Vertical accretion rates and heavy metal chronologies in wetland sediments of the Tijuana Estuary. *Estuaries*, *24*(6), pp.840-850.	
30	Holmquist, James R., Windham-Myers, Lisamarie, Bliss, Norman, Crooks, Stephen, Morris, James T., Megonigal, J. Patrick, Troxler, Tiffany, Weller, Donald E., Callaway, John, Drexler, Judith, Ferner, Matthew C., Gonneea, Meagan E., Kroeger, Kevin D., Schile-Beers, Lisa, Woo, Isa, Buffington, Kevin, Boyd, Brandon M., Breithaupt, Joshua, Brown, Lauren N., Dix, Nicole, Hice, Lyndie, Horton, Benjamin P., MacDonald, Glen M., Moyer, Ryan P., Reay, William et al. 2018. [Dataset] "*Accuracy and Precision of Tidal Wetland Soil Carbon Mapping in the Conterminous United States: Public Soil Carbon Data Release*." Distributed by Smithsonian Research Online.	*
31	Abbott, Katherine M; Quirk, Tracy; Delaune, Ronald D. (2019): Dataset: Factors influencing blue carbon accumulation across a 32-year chronosequence of created coastal marshes. Smithsonian Environmental Research Center. Dataset. https://doi.org/10.25573/data.10005215.v1	
32	Poppe, Katrina L; Rybczyk, John M (2019): Dataset: Sediment carbon stocks and sequestration rates in the Pacific Northwest region of Washington, USA. Smithsonian Environmental Research Center. Dataset. https://doi.org/10.25573/data.10005248.v1	
33	Thom, Ronald M. (2019): Dataset: Accretion rates of low intertidal salt marshes in the Pacific Northwest. Smithsonian Environmental Research Center. Dataset. https://doi.org/10.25573/data.10046189.v2	
34	Callaway, John C.; Borgnis, Evyan L.; Turner, R. Eugene; Milan, Charles S. (2019): Dataset: Carbon sequestration and sediment accretion in San Francisco Bay tidal wetlands. Smithsonian Environmental Research Center. Dataset. https://doi.org/10.25573/data.9693251.v1	
35	Doughty, Cheryl; Langley, J. Adam; Walker, Wayne; Feller, Ilka C.; Schaub, Ronald; Chapman, Samantha (2019): Mangroves marching northward: the impacts of rising seas and temperatures on ecosystems at Kennedy Space Center. Smithsonian Environmental Research Center. Dataset. https://doi.org/10.25573/data.9695918.v1	
36	Boyd, Brandon; Sommerfield, Christopher K.; Quirk, Tracy; Unger, Viktoria (2019): Dataset: Accretion and sediment accumulation in	

	impounded and unimpounded marshes in the Delaware Estuary and Barnegat Bay. Smithsonian Environmental Research Center. Dataset. https://doi.org/10.25573/data.9747065.v1	
37	Breithaupt, Joshua L.; Smoak, Joseph M.; Bianchi, Thomas S.; Vaughn, Derrick; Sanders, Christian J.; Radabaugh, Kara R.; et al. (2020): Dataset: Increasing rates of carbon burial in southwest Florida coastal wetlands. Smithsonian Environmental Research Center. Dataset. https://doi.org/10.25573/serc.9894266.v1	*
38	Vaughn, Derrick; Bianchi, Thomas; Shields, Michael; Kenney, William; Osborne, Todd (2020): Dataset: Increased Organic Carbon Burial in Northern Florida Mangrove-Salt Marsh Transition Zones. Smithsonian Environmental Research Center. Dataset. https://doi.org/10.25573/serc.10552004	
39	Peck, Erin; Wheatcroft, Robert; Brophy, Laura (2019): Dataset: Controls on sediment accretion and blue carbon burial in tidal saline wetlands: Insights from the Oregon coast, U.S.A.. Smithsonian Environmental Research Center. Dataset. https://doi.org/10.25573/serc.11317820.v2	
40	McTigue, Nathan; Davis, Jenny; Rodriguez, Antonio; McKee, Brent; Atencio, Anna; Currin, Carolyn (2020): Dataset: Carbon accumulation rates in a salt marsh over the past two millennia. Smithsonian Environmental Research Center. Dataset. https://doi.org/10.25573/serc.11421063.v1	*
41	Kemp, Andrew C.; Sommerfield, Christopher K.; Vane, Christopher H.; P. Horton, Benjamin; Chenery, Simon; Anisfeld, Shimon; et al. (2020): Dataset: Use of lead isotopes for developing chronologies in recent salt-marsh sediments. Smithsonian Environmental Research Center. Dataset. https://doi.org/10.25573/serc.11569419.v1	
42	Messerschmidt, Tyler C.; Kirwan, Matthew L. (2020): Dataset: Soil properties and accretion rates of C3 and C4 marshes at the Global Change Research Wetland, Edgewater, Maryland. Smithsonian Environmental Research Center. Dataset. https://doi.org/10.25573/serc.11914140.v1	
43	Nolte, Stefanie (2020): Dataset: Does livestock grazing affect sediment deposition and accretion rates in salt marshes?. Smithsonian Environmental Research Center. Dataset. https://doi.org/10.25573/serc.11958996.v1	
44	Lagomasino, David; Corbett, D. Reide; Walsh, J.P. (2020): Dataset: Influence of Wind-Driven Inundation and Coastal Geomorphology on Sedimentation in Two Microtidal Marshes, Pamlico River Estuary, NC. Smithsonian Environmental Research Center. Dataset. https://doi.org/10.25573/serc.12043335.v1	
45	Boone Kauffman, J.; Giovannoni, Leila R.; Kelly, James; Dunstan, Nicholas; Borde, Amy; Diefenderfer, Heida; et al. (2020): Dataset: Carbon stocks in seagrass meadows, emergent marshes, and forested tidal swamps of the Pacific Northwest. Smithsonian Environmental Research Center. Dataset. https://doi.org/10.25573/serc.12640172.v2	
46	Keshta, Amr E.; Yarwood, Stephanie A.; Baldwin, Andrew H. (2020): Dataset: Soil Redox and Hydropattern control Soil Carbon Stocks	

	across different habitats in Tidal Freshwater Wetlands in a Sub-estuary of the Chesapeake Bay. Smithsonian Environmental Research Center. Dataset. https://doi.org/10.25573/serc.13187549.v2	
47	Windham-Myers, L., Marvin-DiPasquale, M.C., Agee, J.L., Kieu, L.H., Kakouros, E., Erikson, L. and Ward, K., 2010. Biogeochemical processes in an urban, restored wetland of San Francisco Bay, California, 2007–2009; methods and data for plant, sediment and water parameters. *US Geological Survey Open-File Report*, *1299*, p.23.	
48	Piazza, S.C., Steyer, G.D., Cretini, K.F., Sasser, C.E., Visser, J.M., Holm, G.O., Sharp, L.A., Evers, D. and Meriwether, J.R., 2011. *Geomorphic and ecological effects of Hurricanes Katrina and Rita on coastal Louisiana marsh communities*. U. S. Geological Survey.	
49	Nyman, J.A., DeLaune, R.D., Roberts, H.H. and Patrick Jr, W.H., 1993. Relationship between vegetation and soil formation in a rapidly submerging coastal marsh. *Marine Ecology Progress Series*, pp.269-279.	
50	Craft, C., 2007. Freshwater input structures soil properties, vertical accretion, and nutrient accumulation of Georgia and US tidal marshes. *Limnology and oceanography*, *52*(3), pp.1220-1230.	
51	Krauss, K.W., Noe, G.B., Duberstein, J.A., Conner, W.H., Jones, M.C., Bernhardt, C.E., Cormier, N., and From, A.S., 2018, Carbon budget assessment of tidal freshwater forested wetland and oligohaline marsh ecosystems along the Waccamaw and Savannah rivers, U.S.A. (2005-2016) (version 2.0, May 2019): U.S. Geological Survey data release, https://doi.org/10.5066/F7TM7930.	
52	Nahlik, A.M. and Fennessy, M., 2016. Carbon storage in US wetlands. *Nature Communications*, *7*(1), pp.1-9.	

References

1. Mcowen, C.J., Weatherdon, L.V., Van Bochove, J., Sullivan, E., Blyth, S., Zockler, C., et al. (2017). A global map of saltmarshes. Biodiversity data journal.
2. Olofsson, P., Foody, G. M., Stehman, S. V. & Woodcock, C. E. Making better use of accuracy data in land change studies: Estimating accuracy and area and quantifying uncertainty using stratified estimation. *Remote Sens. Environ.* **129**, 122-131 (2013).
3. Chen B, Xu B, Zhu Z, Yuan C, Suen HP, Guo J, et al. Stable classification with limited sample: Transferring a 30-m resolution sample set collected in 2015 to mapping 10-m resolution global land cover in 2017. *Sci Bull*. 2019;**64**: 370-373.
4. Huang, L. *et al*. Two-decade wetland cultivation and its effects on soil properties in salt marshes in the Yellow River Delta, China. *Ecological Informatics* **10**, 49-55 (2012)
5. An, S. *et al*. China's natural wetlands: past problems, current status, and future challenges. *AMBIO: A Journal of the Human Environment* **36**, 335-342 (2007).

6. Chen, G., Jin, R., Ye, Z., Li, Q., Gu, J., Luo, M., Luo, Y., Christakos, G., Morris, J., He, J. and Li, D., 2022. Spatiotemporal Mapping of Salt Marshes in the Intertidal Zone of China during 1985–2019. *Journal of Remote Sensing*, *2022*.
7. Bunting, P., Rosenqvist, A., Lucas, R.M., Rebelo, L., Hilarides, L., Thomas, N., Hardy, A., Itoh, T., Shimada, M. & Finlayson, C.M. 2018, "The global mangrove watch—a new 2010 global baseline of mangrove extent", *Remote Sensing,* vol. 10, no. 10, pp. 1669.
